# Characterization of the mitochondrial genome of a high royal jelly-producing honeybee strain (*Apis mellifera ligustica*)

**DOI:** 10.1080/23802359.2021.1974320

**Published:** 2021-09-15

**Authors:** Chuan Ma, Jianke Li

**Affiliations:** Institute of Apicultural Research/Key Laboratory of Pollinating Insect Biology, Ministry of Agriculture, Chinese Academy of Agricultural Sciences, Beijing, China

**Keywords:** Mitochondrial genome, royal jelly, Italian bees, *Apis mellifera ligustica*

## Abstract

The complete mitochondrial genome (mitogenome) of a high royal jelly-producing honeybee strain selected from Italian bees (*Apis mellifera ligustica*) in China was determined. The entire mitogenome was 16,467 bp in length with an A + T content of 84.99%. It contained the typical 13 protein-coding genes, 22 transfer RNA genes, two ribosomal RNA genes, and a control region. The order and orientation of these genes were identical to those of other *A*. *mellifera* subspecies sequenced to date. Phylogenetic analysis supported that the high royal jelly-producing honeybee strain was sister to formerly sequenced Italian bees.

Royal jelly, a natural secretion from nurse bees, is a functional food with health-promoting properties for humans (Ahmad et al. 2020). More than 90% of the global royal jelly is produced in China. This is mainly attributed to the high royal jelly-producing honeybee strain (RJB), which has been genetically bred from Italian bees (ITBs, *Apis mellifera ligustica*) in China since 1980s (Altaye et al. 2019). Relative to ITBs, RJBs could produce over 10-fold more royal jelly (Hu et al. 2019). To contribute to the understanding of the genetic basis of the high royal jelly yield, the complete mitogenome of RJBs was sequenced in this study.

Drones of RJBs were collected from Pinghu, Zhejiang, China (30.682°N, 121.023°E). Whole genomic DNA was extracted from the head of a single drone using a DNeasy Blood and Tissue kit (Qiagen). The tissue and DNA samples (accession number PHB02) were deposited in the entomological specimen room in the Institute of Apicultural Research, Chinese Academy of Agricultural Sciences. High-throughput sequencing was performed on the HiSeq 2500 platform (Illumina Inc.). The full mitogenome was assembled with MITObim v1.9 (Hahn et al. 2013) and annotated on the MITOS webserver (Bernt et al. 2013). The control region sequence was validated via PCR amplification followed by Sanger sequencing. The RJB mitogenome was deposited in GenBank under the accession number MT859135.

The complete RJB mitogenome was a circular molecule of 16,467 bp in size with a strong A + T-biased nucleotide composition (43.29% A, 41.70% T, 9.52% C, and 5.48% G). It possessed a control region and encoded a typical set of 37 genes, that is, 13 protein-coding genes, 22 transfer RNA genes, and two ribosomal RNA genes, as in other insect mitogenomes. The gene order and orientation were identical to those of other *A*. *mellifera* subspecies deposited in GenBank. ATN served as start codons of all protein-coding genes except *atp6* starting with TTG. Incomplete stop codons (T or TA) immediately followed by a transfer RNA gene were adopted by *nad2*, *cox1*, and *cox2*. The 22 transfer RNA genes varied in length from 61 to 78 bp. The control region was 919 bp in length with a high A + T content of 96.30%. Twenty-four intergenic spacers, totaling 844 bp, were detected, with the largest one (193 bp) located between *trnL2* and *cox2*.

Phylogenetic relationships of the RJB and other *A*. *mellifera* subspecies were reconstructed in MrBayes v3.2 (Ronquist et al. 2012) based on concatenated sequences of the 13 protein-coding genes and two ribosomal RNA genes. The Bayesian inference tree (Figure 1) recovered the monophyly of all *A*. *mellifera* subspecies. The newly sequenced RJB was sister to a formerly sequenced ITB (Crozier and Crozier 1993) with a high posterior probability of 1.00. The sister relationship supports that RJBs were genetically bred from ITBs (Altaye et al. 2019). Comparison of the two mitogenome sequences revealed 23 variable sites and 152-bp insertion/deletions with the largest insertion of 80 bp in the control region of the RJB. Among them, eight variable sites including three nonsynonymous ones were located in the 13 protein-coding genes. Further population genetic studies are needed to examine whether the variable sites are fixed in the RJBs.

**Figure 1.**
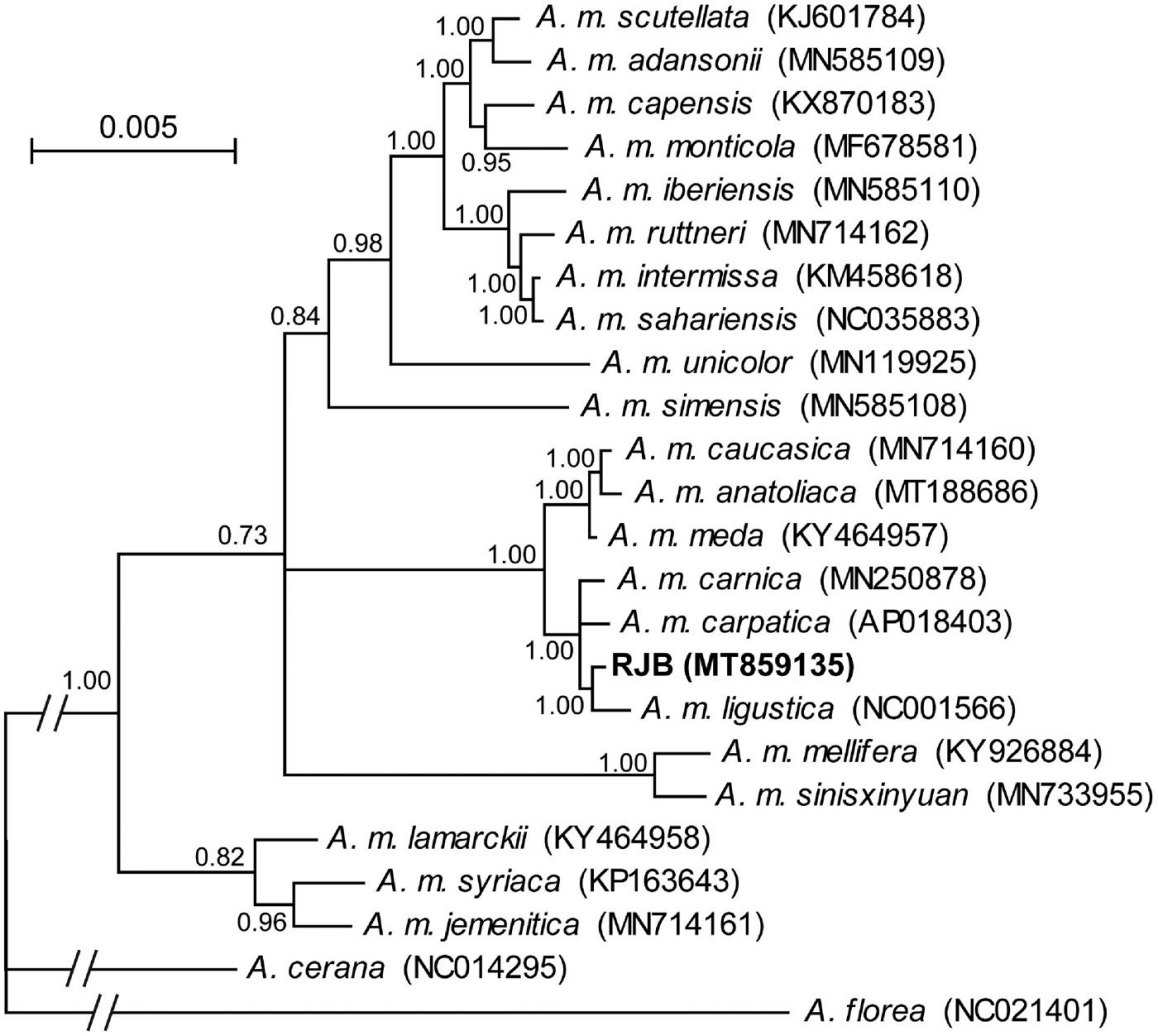
The Bayesian inference tree of *A*. *mellifera* subspecies based on the 13 protein-coding genes and two ribosomal RNA genes. The branches of the outgroup taxa, *A*. *florea* and *A*. *cerana*, are truncated as indicated by slashes. Bayesian posterior probability values are shown at nodes. GenBank accession numbers for each taxon are provided in parenthesis.

## Data Availability

The data that support the findings of this study are openly available in GenBank of NCBI at https://www.ncbi.nlm.nih.gov, reference number MT859135.
